# Modeling Seasonal Influenza Outbreak in a Closed College Campus: Impact of Pre-Season Vaccination, In-Season Vaccination and Holidays/Breaks

**DOI:** 10.1371/journal.pone.0009548

**Published:** 2010-03-04

**Authors:** Kristin L. Nichol, Kate Tummers, Alanna Hoyer-Leitzel, Jennifer Marsh, Matt Moynihan, Steven McKelvey

**Affiliations:** 1 Research Service, Veterans Affairs Medical Center, Minneapolis, Minnesota, United States of America; 2 Department of Medicine, University of Minnesota, Minneapolis, Minnesota, United States of America; 3 Department of Mathematics, Statistics, and Computer Science, St. Olaf College, Northfield, Minnesota, United States of America; Albert Einstein College of Medicine, United States of America

## Abstract

**Background:**

College and university students experience substantial morbidity from influenza and influenza-like illness, and they can benefit substantially from vaccination. Public health authorities encourage vaccination not only before the influenza season but also into and even throughout the influenza season. We conducted the present study to assess the impact of various vaccination strategies including delayed (i.e., in-season) vaccination on influenza outbreaks on a college campus.

**Methods/Findings:**

We used a Susceptible → Infected → Recovered (SIR) framework for our mathematical models to simulate influenza epidemics in a closed, college campus. We included both students and faculty/staff in the model and derived values for the model parameters from the published literature. The values for key model parameters were varied to assess the impact on the outbreak of various pre-season and delayed vaccination rates; one-way sensitivity analyses were conducted to test the sensitivity of the model outputs to changes in selected parameter values. In the base case, with a pre-season vaccination rate of 20%, no delayed vaccination, and 1 student index case, the total attack rate (total percent infected, TAR) was 45%. With higher pre-season vaccination rates TARs were lower. Even if vaccinations were given 30 days after outbreak onset, TARs were still lower than the TAR of 69% in the absence of vaccination. Varying the proportions of vaccinations given pre-season versus delayed until after the onset of the outbreak gave intermediate TAR values. Base case outputs were sensitive to changes in infectious contact rates and infectious periods and a holiday/break schedule.

**Conclusion:**

Delayed vaccination and holidays/breaks can be important adjunctive measures to complement traditional pre-season influenza vaccination for controlling and preventing influenza in a closed college campus.

## Introduction

Influenza is a major cause of morbidity and mortality and each year causes tens of millions of illnesses, hundreds of thousands of excess hospitalizations, and tens of thousands of excess deaths in the US. [1] Vaccination remains the mainstay of efforts to prevent and control influenza, and in the US current recommendations for the use of influenza vaccines encourage all people to receive vaccination. About ¾ of the population is also included in specific high priority groups. [2]

While not included among the high priority groups for vaccination, college and university students may be at increased risk for influenza and influenza-like illnesses (ILIs). Outbreaks on campuses with high attack rates have been described. [3] [4] [5] [6] Furthermore, students experience substantial morbidity from influenza and ILIs. On average they experience up to 8 days or more of illness along with increased rates of health care use, school absenteeism, and impaired academic performance for each ILI. [7] [8] According to national survey data from the American College Health Association, colds/flu/sore throat is the second leading cause of impediments to academic performance. [9] Influenza vaccination has been associated with significant reductions in ILI as well as ILI-associated impaired school performance and health care utilization, [10] and many college and university student health service programs have implemented influenza vaccination programs for their students and faculty.

Influenza vaccination programs, including those on college and university campuses, traditionally have been organized around vaccine delivery in October and November, strategies consistent with many years' advice addressing the timing of vaccination programs from national authorities. However, the Centers for Disease Control and Prevention and others are now calling on providers to expand vaccination efforts into and even throughout the entire influenza season. [2] [11] [12]This strategy is urged in order to take advantage of opportunities to vaccinate people who might otherwise fail to receive their vaccine and to ensure demand for vaccine that might not become available until December or January. Because influenza virus activity–while it often begins in November or December–may not peak until February or March, such a strategy is reasonable. In fact, since 1976>80% of influenza seasons have peaked in January or later with >60% peaking in February or later. [2]

For optimal protection, however, people should be vaccinated before the onset of influenza activity to ensure sufficient time to develop a protective immune response to vaccination. Extending the vaccination period may therefore result in increased numbers of persons vaccinated after the epidemic has started. How this might affect the overall attack rates and the time course of seasonal influenza outbreaks within closed populations such as college campuses has not been well described.

We conducted the present study to model seasonal influenza outbreaks in a closed, college campus setting and to explore the impact of various vaccination scenarios, including vaccination extending into the influenza season, as well as the impact of a holiday/break schedule on these influenza outbreaks.

## Methods

To construct our influenza outbreak model, we adapted selected characteristics of St. Olaf College in Northfield, Minnesota, a residential liberal arts college that has previously participated in a study of the burden of influenza-like illness among college and university students. On the college campus there are about 3000 students and 450 faculty and staff during the academic year. The school is a residential school, and students are required to live in college-owned residential facilities including one of the 10 on-campus co-educational dormitories or in one of the 18 campus houses for upper class students. The dormitories have shared bathrooms, and the college has a single, common cafeteria which facilitates mingling of students and limits the ability of students to withdraw from the community even when they are ill.

### Mathematical Models

For this study, we constructed mathematical models based on the Susceptible-Infectious-Recovered (SIR) framework for epidemiological systems in which the host population is categorized according to infection status. [13] People can move through the various states from susceptible to infectious to recovered. The model assumes that once infected a person is infectious, and that once recovered the person is immune for the rest of that influenza season.

The basic SIR model can be described by 3 differential equations:
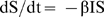


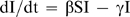


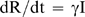



Where S  =  susceptible, I  =  infected, R  =  recovered, β  =  infectious contact rate, γ  =  recovery rate, and 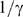  =  infectious period.

**Figure 1:**
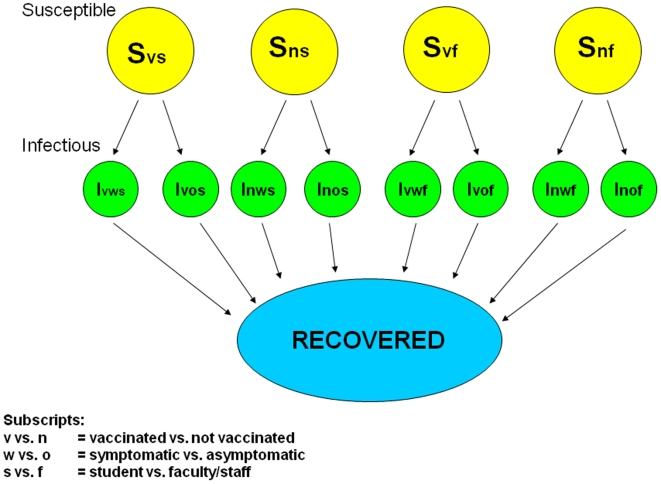
Structure of SIR model. Shown are the various population compartments as people move through the Susceptible → Infected → Recovered states. Yellow denotes susceptible, green infected and blue recovered.

In constructing our models, we stratified the population using several key assumptions. We assumed higher rates of social mixing and therefore higher infectious contact rates among students than among faculty/staff, that vaccination would prevent infection in most vaccinated persons and would attenuate illness among the rest if they became infected, and that infected persons who are asymptomatic have a shorter infectious period and lower infectious contact rate than persons with symptomatic illness. These key assumptions were used to stratify the susceptibles by faculty/staff vs student and by vaccination status. Likewise, among infected persons we stratified by faculty/staff vs student, by vaccination status, and by whether the person was symptomatic vs asymptomatic. The basic structure of the models that we used is shown in figure 1. As can be seen, the basic model has 13 different states (4 susceptible states, 8 infected states, and 1 recovered state).

### Infectious Contact Rates

The key variables for the models are the infectious contact rates and infectious periods for each of the population compartments. Infectious contact rates are a function of social mixing patterns and transmission probabilities given a social contact (ie infectious contract rate  =  # daily contacts * transmission probability given a contact). In a study of a convenience sample of students and staff from two British universities, participants reported about 17 contacts per day. [14] In a study of undergraduate students from the University of Warwick, the students reported about 26 contacts per day during the week and 19 per day on weekends. [15] In another study from two Belgian universities (83.6% of subjects were students) participants reported 18.1 contacts per day during the week and 12.3 on the weekend. [16] For our study we assumed that students would have about 17 social contacts per day. Daily transmission probabilities between infected and susceptible household contacts have been estimated to be 0.025 to 0.087. [17] For our study we assumed a transmission probability of 0.03 given a contact between an infected, unvaccinated person and a susceptible, unvaccinated person. Thus, for an unvaccinated student who becomes ill with symptomatic influenza, we used an infectious contact rate of 0.5 per day (ie 17 contacts per day * 0.03 infections per contact).

Infectious contact rates may be lower among older adults such as faculty than among the students. The social mixing patterns in a population-based study from Belgium suggested that adults 60 and older had about half of the daily or weekly social contacts as persons 13 to 19 years of age. [18] [19] In another study, secondary attack rates within households with an adult index case were about 62% of those where the index case was a school-aged child. [20] For our study we assumed that the infectious contact rate for the faculty/staff would be 60% of that for the students.

### Infectious Periods

A recent review of human influenza virus challenge studies found that viral shedding increased sharply between 12 and 24 hours after viral inoculation and tended to peak on the second day after challenge. The average duration of viral shedding was nearly 5 days, although longer durations of shedding were not rare. [21] Because of the sharp increase in viral shedding within the first 24 hours after infection, we assumed that there was no latent period for our model. We also assumed that the duration of infectiousness was 5 days among unvaccinated persons who became ill, with the same duration for both faculty/staff and students.

### Symptomatic and Asymptomatic Infection

The review of influenza challenge studies also found that upper respiratory symptoms occurred in 58.8% of subjects whereas any symptom occurred in 66.7% of subjects. [21] Other modeling studies have assumed that 50% to 70% of persons will become symptomatic. [21] For our study we assumed that 65% of infected persons would become symptomatic.

The relationship between symptoms and infectiousness has not been well established, although there is some evidence that the presence of symptoms is positively correlated with higher viral shedding titers in experimental human influenza infection. [21] Higher viral shedding titers would therefore be expected to correlate with higher infectious contact rates. We also assumed for the base case that symptomatic people did not withdraw from the community because students living on a residential campus have very limited options for withdrawal. They share dormitory rooms with roommates, use common bathrooms, and eat in a single cafeteria that facilitates mingling between community members. Therefore, for our study we assumed that persons with asymptomatic infection would have half the infectious contact rate of symptomatic persons.

The mean time after infection to the onset of symptoms in influenza virus challenge studies was found to be 1.7 days. [21] In community models of the spread of influenza, the asymptomatic or latent period was estimated to be 1.9 days. [22] For our study we assumed that the total duration of shedding among asymptomatic persons would be limited to 1.9 days.

### Vaccine Efficacy

During years with a good virus-vaccine strain match, influenza vaccination has an efficacy of about 80% for preventing influenza illness in healthy adults under age 65. [23] For our study we assumed that vaccination would reduce the likelihood of infection by 80%.

Even when vaccination does not prevent infection, it may nevertheless result in milder illness. [24] For the 20% of persons for whom vaccination did not prevent infection in our study (ie vaccine failures), we assumed that vaccination would still attenuate the effects of infection by reducing the duration of infectiousness by about 1 day to 4 days for symptomatic persons and to 1 day for asymptomatic persons.

### Model Scenarios

In the base case, we assumed that the campus community was a closed community, that a single symptomatic student introduced influenza onto the campus, that 20% of the population was vaccinated prior to the onset of the influenza season, and that no vaccine was given after the onset of the outbreak. Other scenarios were constructed with varying levels of pre-season (ie vaccination before the onset of the influenza outbreak) and delayed, or in-season, vaccination (ie vaccination after the onset of the influenza outbreak). For the delayed vaccination scenarios, we assumed that vaccinations occurred either 30 or 42 days after the onset of the outbreak.

Additional sensitivity analyses explored the sensitivity of the base case scenario to changes in the values of key model parameters including the number of index cases, duration of infectious periods, and infectious contact rates. Other sensitivity analyses explored the impact of varying vaccination rates according to faculty vs student status. We also modeled the impact the school's typical holiday break schedule on the influenza outbreak. For this scenario we incorporated three school holidays occurring on days 22–26, 51–62, and 89–96 of the outbreak. For the school holiday scenario, we assumed a pre-season vaccination rate of 20% and no delayed vaccination with a single infected, unvaccinated student as the index case. For each break we assumed that all persons who were infected at the beginning of the break would recover before returning to campus because the shortest break was 5 days, the length of the infectious period used in the model. We also assumed that 5 infected and symptomatic unvaccinated students would return from each break, thereby reintroducing influenza into the community. All other parameters were the same as for the base case.

For the base case and each of the sensitivity analysis scenarios, we used deterministic models with fixed parameter values to estimate the epidemic curves, time to the peak day of the outbreak, and total attack rate (ie total percent infected, TAR) during the outbreak. To estimate uncertainty around the deterministic model outputs, for the base case we also constructed a stochastic parameters model. For this model we used the same 13 states and differential equations as in the deterministic model. At each time step (set at 0.1 days) the model randomly sampled one value from a probability distribution for each of the 17 parameters (eight β's, eight γ 's and one Φ [proportion of infected people who are symptomatic]).which were assumed to have a normal distribution with the mean used in the deterministic models and a standard deviation of 20%. 1000 simulations for 200 day periods were performed.

Deterministic models may provide misleading results for small populations in part because they do not account for the discrete nature of populations and the impact that chance events can have on model outputs. We therefore also constructed another, discrete population stochastic model – a continuous-time Markov chain model [25] in which individuals transition through the various possible S-I-R states at randomly assigned times based on conditional probabilities that a given state change will occur. Model parameters were based on the base case assumptions used in the deterministic models. The outputs from this stochastic model after 1000 simulations were therefore used to validate the outputs from the deterministic models. All analyses were conducted using Maple 10 software (Maplesoft, Ontario, Canada). Key base case parameter values are summarized in table 1. Details of the 13 differential equations used in the deterministic models as well as the general equations used to calculate the transition probabilities, conditional probabilities, and inter event times for the discrete population stochastic model are provided in appendix S1.

**Table 1:** Key infection parameter values.*

	Infectious period, days (1/γ)	Infectious contact rate (No. infected contacts per day of infectiousness) (β)
Unvaccinated students		
Symptomatic illness	5	0.5
Asymptomatic infection	1.9	0.25
Unvaccinated faculty		
Symptomatic illness	5	0.3
Asymptomatic infection	1.9	0.15
Vaccinated students		
Symptomatic illness	4	0.5
Asymptomatic infection	1	0.25
Vaccinated faculty		
Symptomatic illness	4	0.3
Asymptomatic infection	1	0.15

*For the models we assumed that vaccine efficacy was 80% for preventing illness, that 65% of infected persons were symptomatic, that asymptomatic persons would be about half as infectious as symptomatic persons with a shorter infectious period, and that faculty would be less infectious than students. We also assumed that the infectious period duration would be reduced among vaccine failures. See the methods section for additional details and references supporting these assumptions for the models' parameter values.

## Results

### Base Case Analyses

In the base case, with a 20% pre-season vaccination rate, the deterministic model predicted a TAR of 45%, an outbreak peak on day 68, and a total outbreak period of 157 days. The range for the TAR from the stochastic parameters model was 40% to 51% and the range for the outbreak peak from 52 days to 92 days. Results from the discrete population stochastic model showed that the likelihood of the outbreak propagating within the closed population was 40% with a single infected person as the index case and 88% with 5 infected persons. The modal TAR value given an outbreak was 45%.

### Senstivity Analyses–Varying Pre-Season and In-Season Vaccination Rates

**Figure 2:**
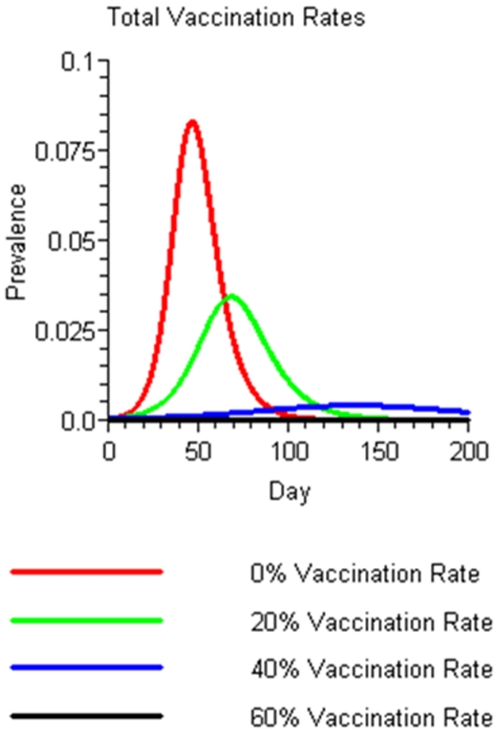
Impact of different pre-season vaccination rates on seasonal influenza outbreak curves. In these scenarios all vaccine was administered pre-season.

With a 0% vaccination rate, the TAR was 69%, with a peak at day 47 and a total outbreak duration of 116 days. In contrast, with a total pre-season vaccination rate of 40%, only 13% of the study population would become infected with the outbreak peak being delayed to 137 days and the total duration of the outbreak extending beyond 200 days. At a 60% vaccination rate <1% became infected suggesting that there was no outbreak. (figure 2)

**Figure 3:**
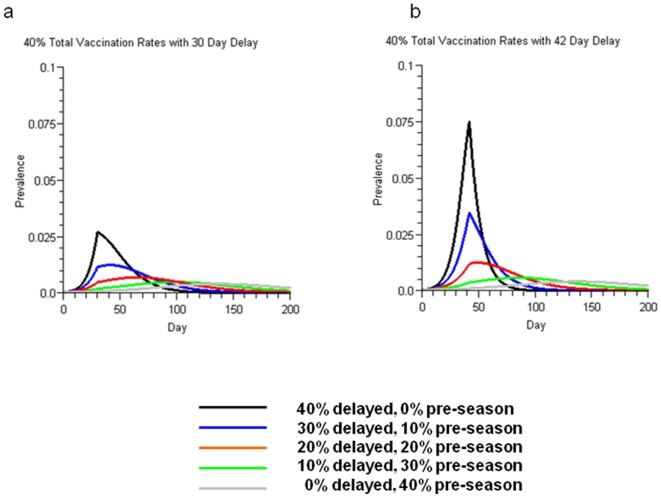
Influenza outbreak curves with varying pre-season and delayed vaccination rates. For all scenarios the total vaccination rate was 40%. Shown are examples with delayed (ie in-season) vaccination occurring 30 days (3a) or 42 days (3b) after the onset of the outbreak. Pre-season vaccination rates  =  40% minus delayed vaccination rate.

The impact of shifting the timing of vaccination to day 30 or 42 after the onset of the outbreak for portions of the population is summarized in table 2. As a larger portion of the total numbers of vaccinations were delayed until after the onset of the outbreak, and as the length of the delay in vaccination increased, then the TARs increased with the outbreak also peaking earlier. Given a total vaccination rate of 40%, the impact of different proportions of vaccinations being given either pre-season or delayed on the shape of the outbreak curve is illustrated in figure 3.

**Table 2:** Impact of varying pre-season, delayed, and total vaccination rates on influenza outbreaks.*

Total % Vacc	Pre- Season Vacc, %	Delayed Vacc, %	In-Season Delay (days)	Total Attack Rate, %	Peak Day of Outbreak	Outbreak Duration (days)
0%	0%	0%	0	69%	47	116
20%	20%	0%	0	45%	68	157
	10%	10%	30	46%	57	144
			42	47%	52	135
	0%	20%	30	47%	45	128
			42	52%	40	110
40%	40%	0%	0	13%	137	>200
	20%	20%	30	18%	65	>200
			42	21%	49	167
	30%	10%	30	16%	99	>200
			42	17%	85	>200
	10%	30%	30	21%	42	160
			42	29%	40	122
	0%	40%	30	26%	25	124
			42	40%	39	94
60%	60%	0%	0	<1%	29	21
	30%	30%	30	2%	27	77
			42	4%	39	98
	40%	20%	30	1%	27	57
			42	1%	39	75
	50%	10%	30	<1%%	27	32
			42	<1%	39	43
	20%	40%	30	5%	28	103
			42	10%	40	110
	10%	50%	30	8%	27	109
			42	19%	39	92
	0%	60%	30	14%	27	87
			42	34%	39	79

*Vacc denotes vaccination. Delayed vaccination occurred after the onset of the outbreak Total vaccination rates  =  pre-season + delayed. Outbreak duration was defined as the time from the initial infectious contact to the time when there was <1 infectious person in the population.

**Figure 4:**
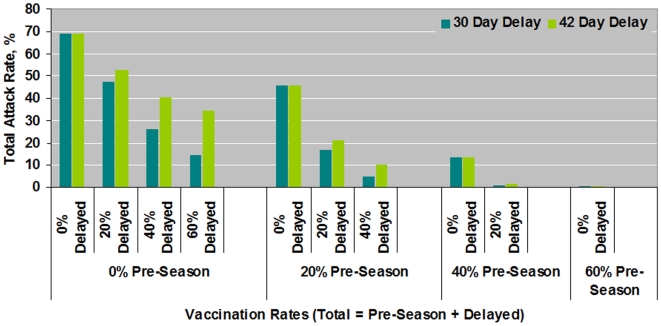
Impact of adding delayed (i.e., in-season) vaccination to pre-season vaccination on total attack rates during influenza outbreaks. Shown are total attack rates for varying levels of pre-season vaccination and delayed (ie in-season) vaccination. In-season vaccination was assumed to occur either 30 or 42 delays after the onset of the outbreak. Total vaccination rates can be calculated by taking the sum of the indicated pre-season rate and delayed vaccination rate.

The addition of delayed vaccination to whatever pre-season vaccination rate is achieved, however, provided additional benefit in further reducing the total numbers of people who will become infected. (figure 4) For example, with a 20% pre-season vaccination rate and no delayed vaccination, the total percent who will become infected is 45%. But with an additional 20% of the population being vaccinated with either a 30 or 42 day delay, the total percentage infected will be reduced to <25%.

Even if there was no pre-season vaccination, delayed vaccination was still of benefit. For example, with no pre-season vaccination and with 20%, 40% or 60% vaccination rates achieved with a 30 day in-season delay, the TAR decreases from 69% with no vaccination at all to 47%, 26%, and 14% respectively (figure 4).

### Additional Sensitivity Analyses

The results of our sensitivity analyses are summarized in table 3. As previously noted, when compared to the base case scenario, as pre-season vaccination rates increased for the population, the outbreak peak was delayed and the total percent infected decreased. The time to the peak of the outbreak and TARs were sensitive to the infectious contact rate and duration of the infectious period. The time to outbreak peak–but not the TAR–was also sensitive to the number of index cases. The model was relatively insensitive to changes in assumptions about the duration of the infectious period and infectious contact rate infections among vaccine failures. The model was also relatively insensitive to proportionately higher vaccination rates among the faculty versus students.

**Table 3:** Sensitivity analyses exploring the impact of changing selected parameter values on the model results.*

	Peak Day	Total Attack Rate, %	Outbreak Duration (days)
**Total vaccination rates (all vaccine pre-season)**			
0%	47	69%	116
20% (base case)	68	45%	157
40%	137	13%	>200
60%	–	<1%	–
**Ratio of Faculty to Student Vaccination Rates** **			
Base case (both at 20%)	68	45%	157
Faculty 30%, student 20%	70	44%	159
Faculty 40%, student 20%	71	43%	162
**No. Index cases**			
1 symptomatic student (base case)	68	45%	157
10 symptomatic students	41	46%	128
20 symptomatic students	33	47%	117
1 symptomatic faculty	74	45%	163
**Infectious Contact Rates (β)**			
Base case	68	45%	157
+10% from base case	55	54%	143
+25% from base case	42	63%	107
−10% from base case	92	34%	>200
−25% from base case	187	5%	>200
**Infectious Contact Rates (β)** ***			
Base case (asymptomatic = 0.5x symptomatic)	68	45%	157
−10% for symptomatic, no change asymptomatic	89	35%	196
−25% for symptomatic, no change for asymptomatic	160	14%	>200
**Infectious Period (1/γ)**			
Base case	68	45%	157
+10% from base case	60	54%	143
+25% from base case	53	63%	133
−10% from base case	83	34%	182
−25% from base case	140	7%	>20
**Attenuation of infection among vaccine failures**			
Base case (infectious period reduced by about 1 day, infectious contact rates same as for unvaccinated persons among vaccine failures)	68	45%	157
Infectious contact rates & infectious periods reduced by 50% among vaccine failures	73	42%	148

*See table 1 for base case parameter values.

**National data suggest that adults >25 years of age tend to have higher vaccination rates than persons 18 to 25 years of age.[see Centers for Disease Control and Prevention, Behavioral Risk Factor Surveillance System data for influenza vaccination for 2005: http://www.cdc.gov/brfss].

***These scenarios were constructed to explore the impact of withdrawal of symptomatic persons with resulting decreases in infectious contact rates on TARs.

### School Break Scenario

The school break scenario incorporated three holiday breaks into the base case scenario (see methods section). Despite the fact that this scenario assumed that, at the end of each break, 5 infected and symptomatic students reintroduced influenza into the community, the TAR was reduced from 45% in the base case without the breaks to only 20% with the breaks.

## Discussion

In this study we have shown how influenza outbreaks in a closed college campus population can be affected by achieving various levels of pre-season, in-season, and total vaccination rates. While optimal control of outbreaks occurs when vaccine is delivered before the onset of the influenza season, we have shown that the addition of delayed vaccination, occurring after the onset of the outbreak, to pre-season vaccination can also be of substantial benefit in reducing total attack rates. Our findings strongly support current recommendations that encourage the expansion of vaccination efforts beyond the traditional October & November window.

The benefits of delayed, in-season vaccination for populations have been described in modeling studies with regard to the control of pandemic influenza when a good portion of vaccine will not be available until after the onset of the pandemic. [26] [27] [28] These studies have shown that delayed availability of vaccine along with other mitigation measures can still contribute substantially to the control of the pandemic. Our study highlights how in-season vaccination should also help to control seasonal influenza–especially in closed settings such as college campuses.

Social distancing strategies such as school closures may also reduce attack rates and help to control influenza outbreaks. In a model based on many years of influenza surveillance data from France, the investigators found that school holidays were associated with reductions of 18%–21% in seasonal influenza illness cases among school children. [29] In another study of elementary school children in Israel, a nationwide temporary closure of elementary schools due to a labor-management dispute resulted in a substantial reduction in respiratory illness rates and healthcare visits among the students. [30] In our study, we also found that school breaks/holidays could have a significant impact on attack rates on the campus. Compared to the base case without breaks or holidays, the introduction of the three school breaks into the model scenario reduced the overall attack rate by about 25%, from 45% to 20%. Our models were not designed to explore how the timing and number of breaks might be optimized for outbreak control, however, and these questions warrant further exploration.

Other mitigation strategies such as isolation, hand hygiene and the use of face masks have been also been proposed for preventing influenza transmission. [26] [28] Clinical trials confirm that such measures may reduce influenza within households, [31] [32] and results from a clinical trial among university students demonstrated a 35% to 51% reduction in the rate of ILI among students who used face masks and enhanced hand hygiene. [33] The findings from our model are consistent with these reports. A 10% reduction in infectious contact rates, for example, reduced the TAR from 45% to 34% whereas a 25% reduction in infectious contact rates reduced the TAR to 5%. These findings highlight the possible benefits of effectively implementing these kinds of mitigation strategies.

The potential impact of herd immunity on the nature of seasonal influenza outbreaks has long been recognized, [34] with a number of recent studies focusing especially on the community-wide [35] and household [36] benefits associated with high vaccination rates of children. In our closed campus models we also demonstrated the potential for herd immunity to benefit the entire campus community. In fact, with a pre-season vaccination rate of about 50%, the influenza outbreaks were not sustained. A practical implication of this finding is that an increase in vaccination rates of only 20% to 30% above current levels might be sufficient to substantially improve the control of influenza outbreaks in closed college campus communities.

Our study findings are based on mathematical models, and they should be interpreted with caution. To enhance the validity of our findings, we derived parameter estimates from the published literature and conducted sensitivity analyses to demonstrate how our results might be affected by changes in the parameter values. We did use deterministic models for most of our analyses which may provide misleading results when analyzing small populations. However, the TAR results from our discrete population stochastic model were in good agreement with our deterministic model results. Good agreement between deterministic and discrete population models with population sizes of 800 or more has previously been reported. [37] Our study population was 3450. Other aspects of our results also lend credibility to our findings. The basic reproductive number for our model was 1.7 for the fully susceptible population. This is consistent with the 1.7 to 2.1 range of basic reproductive numbers recently estimated for seasonal influenza epidemics in temperate regions. [38] In addition, the total attack rates observed in our models are biologically plausible. For example, in our scenario that included the school holiday/break schedule, the model estimated that 20% of the students would become infected during the outbreak corresponding with a 13% clinical influenza rate (65% of 20% being symptomatic). This modeled clinical attack rate is consistent with the 9% to 20% influenza illness rates reported among college and university students in other studies. (10) Given the above, we believe that our findings are likely valid and robust.

### Conclusion

Influenza is a major cause of morbidity on college and university campuses, and vaccination is the primary means available for preventing and controlling influenza outbreaks in these settings. Delayed vaccination occurring after the onset of the outbreak and other mitigation strategies such as holidays/breaks and interventions to reduce infectious contact rates can augment the effectiveness of traditional pre-season vaccination activities for the prevention and control of influenza in these settings.

## Supporting Information

Appendix S1 This appendix provides a detailed description of the equations used in the deterministic models and the discrete population continuous time Markov chain model.
(0.08 MB DOC)

## References

[1] Molinari NA, Ortega-Sanchez IR, Messonnier ML, Thompson WW, Wortley PM (2007). The annual impact of seasonal influenza in the US: measuring disease burden and costs.. Vaccine.

[2] Advisory Committee on Immunization Practices (2008). Prevention and control of influenza. Recommendations of the Advisory Committee on Immunization Practices, ACIP 2008.. MMWR.

[3] Mogabgab WJ (1968). Acute respiratory illnesses in university (1962–1966), military, and industrial (1962–1963) populations.. Am Rev Respir Dis.

[4] Layde PM, Engelberg AL, Dobbs HI, Curtis AC, Craven RB (1980). Outbreak of influenza A/USSR/77 at Marquette University.. J Infect Dis.

[5] Pons VG, Canter J, Dolin R (1980). Influenza A/USSR/77 (H1N1) on a university campus.. Am J Epidemiol.

[6] Sobol J, Loveland FC (1982). Infectious disease in a total institution: a study of the influenza epidemic of 1978 on a college campus.. Public Health Rep.

[7] Nichol KL, D'Heilly S, Ehlinger E (2005). Colds and influenza-like illnesses in university students: impact on health, academic and work performance, and health care use.. Clin Infect Dis.

[8] Nichol KL, D'Heilly S, Ehlinger E (2006). Burden of upper respiratory illnesses among college and university students: 2002–2003 and 2003–2004 cohorts.. Vaccine.

[9] American College Health Association (2006). National College Health Assessment. Reference Group Data Report. Spring 2006.. http://www.acha-ncha.org.

[10] Nichol KL, D'Heilly S, Ehlinger EP (2008). Influenza vaccination among college and university students: impact on influenza-like illness, health care use, and impaired school performance.. Arch Pediatr Adolesc Med.

[11] Orenstein WA, Schaffner W (2008). Lessons learned: role of influenza vaccine production, distribution, supply, and demand–what it means for the provider.. Am J Med.

[12] Poland GA, Johnson DR (2008). Increasing influenza vaccination rates: the need to vaccinate throught the entire influenza season.. Am J Med.

[13] Kermack WO, McKendrick AG (1927). A contribution to the mathematical theory of epidemics.. Proc Roy Soc Lond.

[14] Edmunds WJ, O'Callaghan CJ, Nokes DJ (1997). Who mixes with whom? A method to determine the contact patterns of adults that may lead to the spread of airborne infections.. Proc R soc Lond B.

[15] Edmunds WJ, Kafatos G, Wallinga J, Mossong JR (2006). Mixing patterns and the spread of close-contact infectious diseases.. Emerg Themes Epidemiol.

[16] Beutels P, Shkedy Z, Aerts M, Va Damme P (2006). Social mixing patterns for transmission models of close contact infections: exploring self-evaluation and diary-based data collection through a web-based interface.. Epidemiol Infect.

[17] Longini IM, Seaholm SK, Ackerman E, Koopman JS, Monto AS (1984). Simulation studies of influenza epidemics: assessment of parameter estimation and sensitivity.. Int J Epidemiol.

[18] Wallinga J, Teunis P, Kretzschmar M (2006). Using data on social contacts to estimate age-specific transmission parameters for respiratory-spread infectious agents.. Am J Epidemiol.

[19] Mossong J, Hens N, Jit M, Beutels P, Auranen K (2008). Social contacts and mixing patterns relevant to the spread of infectious diseases.. PLoS Medicine.

[20] Viboud C, Boelle PY, Cauchemez S, Lavenu A, Valleron AJ (2004). Risk factors of influenza transmission within households.. Br J Gen Pract.

[21] Carrat F, Vergu E, Ferguson NM, Lemaitre M, Cauchemex S (2008). Time lines of infection and disease in human influenza: a review of volunteer challenge studies.. Am J Epidemiol.

[22] Elveback LR, Fox JP, Ackerman E, Langworthy A, Boyd M (1976). An influenza simulation model for immunization studies.. Am J Epidemiol.

[23] Jefferson TO, Rivetti D, Di Pietrantonj C, Rivetti A, Demicheli V (2007). Vaccines for preventing influenza in healthy adults.. Cochrane database of systematic reviews.

[24] Couch RB (1993). Advances in influenza virus vaccine research.. Annal NY Acad Sci.

[25] Allen LJS (2003). An introduction to stochastic processes with applications to biology..

[26] Germann TC, Kadau K, Longini IM, Macken CA (2006). Mitigation strategies for pandemic influenza in the United States.. Proc Natl Acad Sci USA.

[27] Carrat F, Luong J, Lao H, Salie AV, Lajaunie C (2006). A ‘small-world-like’ model for comparing interventions aimed at preventing and controlling influenza pandemics.. BMC Med.

[28] Fergusson NM, Cummings DA, Fraser C, Cajka JC, Cooley PC (2006). Strategies for mitigating an influenza pandemic.. Nature.

[29] Cauchemez S, Valleron AJ, Boelle PY, Flahault A, Ferguson NM (2008). Estimating the impact of school closure on influenza transmission from Sentinel data.. Nature.

[30] Heymann A, Chodick G, Reichman B, Kokia E, Laufer J (2004). Influence of school closure on the incidence of viral respiratory diseases among children and on health care utilization.. Ped Infect Dis J.

[31] Jefferson T, Foxlee R, Del Mar C, Dooley L, Ferroni E (2007). Interventions for the interruption or reduction of the spread of respiratory viruses.. Cochrane Database of Systematic Reviews.

[32] Cowline BJ, Chan KH, Cheng CK, Fung RO, Wai W (2009). Facemasks and hand hygiene to prevent influenza transmission in households. A cluster randomized trial.. Ann Intern Med.

[33] Aiello AE, Murray GF, Perez V, Coulborn RM, Davis BM (2010). Mask use, hand hygiene, and seasonal influenza-like illness among young adults: a randomized intervention trial.. J Infect Dis.

[34] Jordan R, Connock M, Albon E, Fry-Smith A, Olowokue B (2006). Universal vaccination of children against influenza: are there indirect benefits to the community? A systematic review of the evidence.. Vaccine.

[35] Piedra PA, Gaglani MJ, Kozinetz CA, Herschler G, Riggs M (2005). Herd immunity in adults against influenza-related illnesses with the use of the trivalent-live attenuated influenza vaccine (CAIV-T) in children.. Vaccine.

[36] King JC, Stoddard JJ, Gaglani MUJ, Moore KA, Magder L (2006). Effectiveness of school-based influenza vaccination.. N Engl J Med.

[37] Xu Y, Allen LJS, Perelson AS (2007). Stochastic model of an influenza epidemic with drug resistance.. J Theor Biol.

[38] Truscott J, Fraser C, Hinsley W, Cauchemez S, Donnelly C (2009). Quantifying the transmissibility of human influenza and its seasonal variation in temperate regions.. Version 3. PLoS Curr Influenza.

