# Supplementary material for: Modeling Seasonal Influenza Outbreak in a Closed College Campus: Impact of Pre-Season Vaccination, In-Season Vaccination and Holidays/Breaks
Source: PLoS One. 2010 Mar 4;5(3):e9548. doi: 10.1371/journal.pone.0009548 (PMC2832017; doi:10.1371/journal.pone.0009548)
Supplement: Appendix S1 — This appendix provides a detailed description of the equations used in the deterministic models and the discrete population continuous time Markov chain model. (0.08 MB DOC) [file pone.0009548.s001.doc]

Technical Appendix

DETERMINISTIC MODELS

Notation

| Notation | SIR Category | Modified Subgroup |
| --- | --- | --- |
| Svs | Susceptible | Vaccinated Students |
| Sns | Susceptible | Non-vaccinated Students |
| Svf | Susceptible | Vaccinated Faculty/Staff |
| Snf | Susceptible | Non-vaccinated Faculty/Staff |
| Ivws | Infectious | Vaccinated, Symptomatic Students |
| Ivos | Infectious | Vaccinated, Asymptomatic Students |
| Inws | Infectious | Non-vaccinated, Symptomatic Students |
| Inos | Infectious | Non-vaccinated, Asymptomatic Students |
| Ivwf | Infectious | Vaccinated, Symptomatic Faculty/Staff |
| Ivof | Infectious | Vaccinated, Asymptomatic Faculty/Staff |
| Inwf | Infectious | Non-vaccinated, Symptomatic Faculty/Staff |
| Inof | Infectious | Non-vaccinated, Asymptomatic, Faculty/Staff |
| R | Recovered |  |

For notation’s sake we kept S, I and R as the main variable and used subscripts to differentiate by vaccination status (v for vaccinated and n for not vaccinated), existence of symptoms (w for with symptoms and o for without symptoms), and students (s) versus faculty/staff (f).

The four susceptible equations can be seen below.

$$\frac{dS_{vs}}{dt}=-(.2)S_{vs}(\beta_{vws}I_{vws}+\beta_{vos}I_{vos}+\beta_{nws}I_{nws}+\beta_{nos}I_{nos}+\beta_{vwf}I_{vwf}+\beta_{vof}I_{vof}+\beta_{nwf}I_{nwf}+\beta_{nof}I_{nof})$$

$\frac{dS_{ns}}{dt}=-S_{ns}(\beta_{vws}I_{vws}+\beta_{vos}I_{vos}+\beta_{nws}I_{nws}+\beta_{nos}I_{nos}+\beta_{vwf}I_{vwf}+\beta_{vof}I_{vof}+\beta_{nwf}I_{nwf}+\beta_{nof}I_{nof}$)

$$\frac{dS_{vf}}{dt}=-(.2)S_{vf}(\beta_{vws}I_{vws}+\beta_{vos}I_{vos}+\beta_{nws}I_{nws}+\beta_{nos}I_{nos}+\beta_{vwf}I_{vwf}+\beta_{vof}I_{vof}+\beta_{nwf}I_{nwf}+\beta_{nof}I_{nof})$$

$$\frac{dS_{nf}}{dt}=-S_{nf}(\beta_{vws}I_{vws}+\beta_{vos}I_{vos}+\beta_{nws}I_{nws}+\beta_{nos}I_{nos}+\beta_{vwf}I_{vwf}+\beta_{vof}I_{vof}+\beta_{nwf}I_{nwf}+\beta_{nof}I_{nof})$$

Each equation represents the rate of change for the different susceptible populations: Svs, Sns, Svf, and Snf (see notation table). There are eight different infectious groups: Ivws, Ivos, Inws, Inos, Ivwf, Ivof, Inwf, and Inof (see notation table) which interact with each susceptible population. Each interaction is described by a β particular to the specific infectious group. The coefficient of .2 in the equations of the susceptible vaccinated populations indicates that twenty percent of the people who are vaccinated are still susceptible. In other words, we made the assumption that our vaccination was 80% effective.

Below are the eight infectious equations. There are eight infectious groups because each of the four susceptible groups can become symptomatic or asymptomatic infectious populations.

$$\frac{dI_{vws}}{dt}=\varphi(.2)S_{vs}(\beta_{vws}I_{vws}+\beta_{vos}I_{vos}+\beta_{nws}I_{nws}+\beta_{nos}I_{nos}+\beta_{vwf}I_{vwf}+\beta_{vof}I_{vof}+\beta_{nwf}I_{nwf}+\beta_{nof}I_{nof})-\gamma_{vws}I_{vws}$$

$$\frac{dI_{vos}}{dt}=(1-\varphi)(.2)S_{vs}(\beta_{vws}I_{vws}+\beta_{vos}I_{vos}+\beta_{nws}I_{nws}+\beta_{nos}I_{nos}+\beta_{vwf}I_{vwf}+\beta_{vof}I_{vof}+\beta_{nwf}I_{nwf}+\beta_{nof}I_{nof})-\gamma_{vos}I_{vos}$$

$$\frac{dI_{nws}}{dt}=\varphi(.2)S_{ns}(\beta_{vws}I_{vws}+\beta_{vos}I_{vos}+\beta_{nws}I_{nws}+\beta_{nos}I_{nos}+\beta_{vwf}I_{vwf}+\beta_{vof}I_{vof}+\beta_{nwf}I_{nwf}+\beta_{nof}I_{nof})-\gamma_{nws}I_{nws}$$

$$\frac{dI_{nos}}{dt}=(1-\varphi)(.2)S_{ns}(\beta_{vws}I_{vws}+\beta_{vos}I_{vos}+\beta_{nws}I_{nws}+\beta_{nos}I_{nos}+\beta_{vwf}I_{vwf}+\beta_{vof}I_{vof}+\beta_{nwf}I_{nwf}+\beta_{nof}I_{nof})-\gamma_{nos}I_{nos}$$

$$\frac{dI_{vwf}}{dt}=\varphi(.2)S_{vf}(\beta_{vws}I_{vws}+\beta_{vos}I_{vos}+\beta_{nws}I_{nws}+\beta_{nos}I_{nos}+\beta_{vwf}I_{vwf}+\beta_{vof}I_{vof}+\beta_{nwf}I_{nwf}+\beta_{nof}I_{nof})-\gamma_{vwf}I_{vwf}$$

$$\frac{dI_{vof}}{dt}=(1-\varphi)(.2)S_{vf}(\beta_{vws}I_{vws}+\beta_{vos}I_{vos}+\beta_{nws}I_{nws}+\beta_{nos}I_{nos}+\beta_{vwf}I_{vwf}+\beta_{vof}I_{vof}+\beta_{nwf}I_{nwf}+\beta_{nof}I_{nof})-\gamma_{vof}I_{vof}$$

$$\frac{dI_{nwf}}{dt}=\varphi(.2)S_{nf}(\beta_{vws}I_{vws}+\beta_{vos}I_{vos}+\beta_{nws}I_{nws}+\beta_{nos}I_{nos}+\beta_{vwf}I_{vwf}+\beta_{vof}I_{vof}+\beta_{nwf}I_{nwf}+\beta_{nof}I_{nof})-\gamma_{nwf}I_{nwf}$$

$$\frac{dI_{nof}}{dt}=(1-\varphi)(.2)S_{nf}(\beta_{vws}I_{vws}+\beta_{vos}I_{vos}+\beta_{nws}I_{nws}+\beta_{nos}I_{nos}+\beta_{vwf}I_{vwf}+\beta_{vof}I_{vof}+\beta_{nwf}I_{nwf}+\beta_{nof}I_{nof})-\gamma_{nof}I_{nof}$$

The Φ term in the equations represents the proportion of infected people who are symptomatic. The (1- Φ) term is, in effect, the proportion of infected people who are asymptomatic. The interaction between the susceptible and infectious categories are represented in the same way as they appear in the susceptible equation. Because there are also people leaving each of the infectious categories, there is a γI term, which signifies the rate of recovery. Note that each γ is specific to its corresponding infectious group.

The recovered equation is a collection of all the people moving out of the infectious categories.

$$\frac{dR}{dt}=\gamma_{vws}I_{vws}+\gamma_{vos}I_{vos}+\gamma_{nws}I_{nws}+\gamma_{nos}I_{nos}+\gamma_{vwf}I_{vwf}+\gamma_{vof}I_{vof}+\gamma_{nwf}I_{nwf}+\gamma_{nof}I_{nof})$$

The rate of change of the recovered category is equal to the sum of the rates of change of the recovery term for every infectious group.

DISCRETE POPULATION CONTINUOUS TIME MARKOV CHAIN MODEL

General equations used to calculate transition probabilities, state change probabilities (conditional probabilities for each state change given that a state change too place) and inter event time. In addition to previously defined notations, P denotes probability, and N denotes number in that state. T denotes the inter event time and Y is a uniform random variable on [0,1]. Given an initial condition, two randomly selected variables are selected: the inter event time and the state change that occurs.

Transition Probabilities

$$P(S\to I)=\frac{\beta}{N}S(t)I(t)\Delta t$$

$$P(I\to R)=\gamma I(t)\Delta t$$

$$P(\text{No Change})=1-(\frac{\beta}{N}S(t)I(t)\Delta t+\gamma I(t)\Delta t)$$

State Change Probabilities

$$P(S\to I|\text{ State Change})=\frac{\frac{\beta}{N}S(t)I(t)\Delta t}{\frac{\beta}{N}S(t)I(t)\Delta t+\gamma I(t)\Delta t}=\frac{\frac{\beta}{N}S(t)}{\gamma+\frac{\beta}{N}S(t)}$$

$$P(I\to R|\text{ State Change})=1-P(S\to I|\text{State Change})=\frac{\gamma}{\gamma+\frac{\beta}{N}S(t)}$$

$$T=-\frac{\ln(Y)}{\frac{\beta}{N}S(t)I(t)+\gamma I(t)}$$
